# Maternal pre-pregnancy BMI, *MTHFR* polymorphisms, and the risk of adverse pregnancy outcomes in pregnant women from South China: a retrospective cohort study

**DOI:** 10.1186/s12884-023-05605-6

**Published:** 2023-04-27

**Authors:** Chunming Gu, Weixiang Wu, Kefeng Lai, Huan Li, Lihong Wu, Weiming Lu, Xiaolin Ruan, Mingyong Luo

**Affiliations:** Department of Clinical Laboratory, Guangdong Women and Children Hospital, Guangzhou, China

**Keywords:** Methylenetetrahydrofolate reductase polymorphisms, Pre-pregnancy BMI, adverse pregnancy outcomes, Gestational hypertension, Gestational diabetes, Cesarean delivery

## Abstract

**Background:**

Increasing evidence suggests an association between maternal pre-pregnancy body mass index (pre-BMI) and adverse pregnancy outcomes. However, the effects of methylenetetrahydrofolate reductase (MTHFR) polymorphisms on these relationships require further investigation. This study aimed to investigate whether the relationship between pre-BMI and the risk of adverse pregnancy outcomes was influenced by *MTHFR* gene polymorphisms.

**Methods:**

A total of 5614 mother-fetus pairs were included in the study. The odds ratios (OR) of adverse pregnancy complications, including gestational diabetes mellitus (GDM), gestational hypertension (GHT), cesarean delivery (CS), and premature rupture of membranes (PROM), were estimated using adjusted logistic regression models and subgroup analysis.

**Results:**

Pregnant women with higher pre-BMI values were positively related to the risk of GDM, GHT, and CS. In the subgroup analysis, underweight BMI was associated with a decreased risk of CS and GDM in pregnant women with the *MTHFR* A1298C AA or C677T CC genotype, while overweight/obese BMI was associated with an increased risk of GDM and CS in different *MTHFR* variants. Moreover, pregnant women with *MTHFR* A1298C AC + CC or C667T CC were found to have an increased risk of GHT in the *MTHFR* A1298C AA or C667T CT + TT genotype. A remarkable association was observed between the obesity group with *MTHFR* A1298C AC + CC (OR = 6.49, CI: 2.67–15.79) and the overweight group with the C667T CC genotype (OR = 4.72, CI: 2.13–10.45).

**Conclusions:**

*MTHFR* gene polymorphisms exert a modifying effect on the association between maternal pre-BMI and the risk of GHT, CS, and GDM. Pregnant women with a high pre-BMI with specific *MTHFR* genotypes should be considered for GHT development.

**Supplementary Information:**

The online version contains supplementary material available at 10.1186/s12884-023-05605-6.

## Introduction

Adverse pregnancy outcomes, including gestational hypertension (GHT), gestational diabetes mellitus (GDM), and cesarean section (CS), have received major public concern globally because of the increasing prevalence of overweight or obesity in women of reproductive age [1, 2]. Women with a history of adverse pregnancy complications and their offspring are at long-term risk of developing obesity, diabetes, hypertension, or other metabolic dysfunctions [3–5]. Therefore, it is important to understand the relationship between maternal conditions before pregnancy and pregnancy-related complications.

Studies from different populations have suggested that maternal weight status prior to pregnancy is a good predictor of pregnancy outcomes, and several studies have linked pre-pregnancy body mass index (pre-BMI) to adverse pregnancy complications [6–8]. Although no significant influence was suggested on infant neurocognitive development based on the Chinese BMI category, being overweight or obese before pregnancy was associated with an increased risk of GDM (odds ratio [OR] = 1.80, 95% confidence interval [CI]: 1.29–2.51) and CS (OR = 1.49, 95% CI: 1.07–2.06) [9]. According to a meta-analysis of European, North American, and Australian populations, higher pre-BMI was associated with higher risks of GHT and GDM [10]. These studies suggest that proper weight before pregnancy might decrease the risk of pregnancy complications.

There is increasing concern regarding whether genetic polymorphisms within lipid metabolism or nutritional factors in pregnant women are associated with the development of pregnancy outcomes [11]. Among them, methylenetetrahydrofolate reductase (MTHFR) plays an important role in the enzymatic process for maintaining folate metabolism and homocysteine methylation, which are considered risk factors during pregnancy [12–14]. The C677T and A1298C variants in *MTHFR* are two common single nucleotide polymorphisms (SNPs), and these variants have also been associated with several pregnancy complications, but the results were different. For instance, a meta-analysis based on 51 studies found significant associations between the *MTHFR* C677T polymorphism and the risk of preeclampsia in TT genotype recessive models and the dominant genetic model [15]. A Chinese study reported that the *MTHFR* C677T variant might increase the risk of GDM owing to higher red blood cell folate levels in early pregnancy [16]. Additionally, genetic variation in *MTHFR* C677T was also related to overweight and obesity in Chinese women, and serum triglyceride levels in the *MTHFR* C677T TT genotype were higher than those in the C677T CC genotype [17].

Although the impact of being overweight or obese is considered strongly related to pregnancy-induced complications, the effect of genetic variations in *MTHFR* on these relationships remains unclear. Therefore, this study was performed to investigate the association between pre-BMI, *MTHFR* C677T and A1298C polymorphisms, and adverse pregnancy outcomes in pregnant Chinese women.

## Materials and methods

### Study population

In the present retrospective cohort study, we included pregnant women who had established regular prenatal records and intended to deliver at Guangdong Women and Children Hospital (Guangzhou, China) between January 2020 to December 2021, according to the following inclusion criteria: (1) > 18 years old, (2) singleton pregnancy, and (3) complete data on basic information. Pregnant women were excluded if they (1) had multiple pregnancies (n = 101), (2) had a diagnosis of diabetes before pregnancy (n = 81), or (3) had a diagnosis of hypertension before pregnancy (n = 55) (4) had undergone in vitro fertilization (n = 256), (5) had incomplete weight or height or *MTHFR* gene polymorphism records (n = 482). A total of 5614 mother-fetus pairs were included in this study (Supplementary Materials Figure S1). The study protocol was approved by the Ethics Committee of Guangdong Women and Children Hospital. This study was conducted in accordance with the Declaration of Helsinki, revised in 1983, and the guidelines of the center’s institutional review board. All participants received details regarding the study and provided written informed consent.

Information on participants’ age, smoking habits, drinking habits, gravidity, parity, education level, delivery mode, weight, height, adverse pregnancy outcomes, and homocysteine levels was collected from the medical records of our hospital. The main outcomes were common adverse pregnancy complications as follows: (1) GDM (defined according to the International Association of Diabetes and Pregnancy Study Groups and World Health Organization); (2) GHT, (diagnosed according to the International Society for the Study of Hypertension [18]); (3) CS; and (4) PROM. The pre-BMI was calculated as weight (kg)/height (m^2^). According to the Chinese BMI classification [19], participants were divided into four groups, namely, normal weight (BMI = 18.5–24.0 kg/m^2^), overweight (BMI = 24.0–28.0 kg/m^2^), obesity (BMI ≥ 28 kg/m^2^), and underweight (BMI < 18.5 kg/m^2^).

### MTHFR gene polymorphisms

Blood samples were retrieved from the participants using vacuum tubes containing potassium salt of ethylenediaminetetraacetic acid and stored at 4 °C. DNA extraction kits (Magen, Guangzhou, China) were used to extract genomic DNA using an automated nucleic acid extraction workstation (Hamilton, Sweden) according to the manufacturer’s instructions. Genotypes for the *MTHFR* C677T and A1298C loci were determined using fluorescence quantitative polymerase chain reaction. The forward and reverse primer sequences used in this study are listed in Supplementary Materials Table S1.

### Statistical analysis

The baseline characteristics of the study participants are described. Data are presented as mean ± standard deviation for continuous variables or as percentages for categorical variables. Continuous variables were compared using parametric methods for continuous data or non-parametric methods for categorical data. For adverse pregnancy outcomes, multiple logistic regression analyses were performed to estimate the OR and 95% CI according to the Chinese BMI standard and *MTHFR* gene polymorphism. Participants with a normal BMI, *MTHFR* A1298C AA genotype, or C677T CC genotype were used as the reference group. The potential covariates used in this regression model are based on relevant reports. GDM, PROM, and CS were adjusted for maternal age, education level, parity, and homocysteine level. Gestational age at delivery was further adjusted in the models with regard to GHT. Deviation from Hardy-Weinberg expectation (HWE) was calculated, and a *P* > 0.05 indicated that the two variants were in accordance with HWE. For subgroup analysis, the modification effect of *MTHFR* gene polymorphisms on the association of pre-BMI groups in relation to adverse pregnancy outcomes was evaluated after stratification by *MTHFR* A1298C or C667T dominant models. *P* for the interaction was assessed using the likelihood ratio test by introducing the multiplicative interaction terms of genotypes and BMI categories. All statistical analyses were conducted using SPSS 26.0 (Chicago, IL, USA), and two-sided *P* < 0.05 was considered statistically significant.

## Results

### Study population

A total of 5614 mother-infant pairs were enrolled in our retrospective cohort study, and their detailed demographic characteristics are shown in Table 1. In this study, none of the mothers had a history of smoking or drinking during pregnancy, and data were not available. The average maternal age was 30.29 ± 4.19 years. Pre-pregnancy BMIs were grouped according to the Chinese BMI classification. There were 1103 women in the underweight group (19.7%), 4012 in the normal-weight group (71.5%), 386 in the overweight group (6.9%), and 113 in the obese group (2.0%). Most mothers were nulliparous (n = 3263, 58.1%), and 2049 (36.5%) women underwent CS. No significant differences in parity, gestational weeks, homocysteine, *MTHFR* C677T, or A1298C genotype were observed among the four pre-BMI groups. Additionally, 53.5% (n = 3005) were boys and 46.5% (n = 2609) were infants. Among them, 4.86% (n = 273) were preterm births. The mean weight, length, and gestational age at birth were 3192.3 ± 427.9 g, 49.5 ± 2.02 cm, and 39.2 ± 1.39 weeks, respectively.

**Table 1:** Clinical characteristics of the study population

	Underweight	Normal weight	Overweight	Obesity	Total		*P*
	n = 1103	n = 4012	n = 386	n = 113	n = 5614		
**Mothers**							
Pre-pregnancy BMI (kg/m2)	17.50 ± 0.79	21.06 ± 1.64	26.17 ± 0.84	29.91 ± 1.64	20.89 ± 2.82		< 0.001
Maternal age (years)	28.85 ± 3.76	30.56 ± 4.19	31.49 ± 4.36	30.84 ± 4.09	30.29 ± 4.19		< 0.001
Gestational weeks	39.24 ± 1.31	39.22 ± 1.40	39.04 ± 1.61	39.20 ± 1.34	39.21 ± 1.39		0.075
Education level							< 0.001
<High school	121 (10.97%)	459 (11.44%)	67 (17.36%)	22 (19.47%)	669 (11.92%)		
High school	170 (15.41%)	613 (15.28%)	75 (19.43%)	20 (17.7%)	878 (15.64%)		
>High school	812 (73.62%)	2940 (73.28%)	244 (63.21%)	71 (62.83%)	4067 (72.44%)		
Parity							< 0.001
Nulliparous	775 (70.26%)	2275 (56.7%)	152 (39.38%)	61 (53.98%)	3263 (58.12%)		
Secundiparous	299 (27.11%)	1568 (39.08%)	206 (53.37%)	45 (39.82%)	2118 (37.73%)		
Multiparous	29 (2.63%)	169 (4.21%)	28 (7.25%)	7 (6.19%)	233 (4.15%)		
**Infant**							
Infant sex							0.552
Male	579 (52.49%)	2168 (54.04%)	203 (52.59%)	55 (48.67%)	3005 (53.53%)		
Female	524 (47.51%)	1844 (45.96%)	183 (47.41%)	58 (51.33%)	2609 (46.47%)		
Birthweight	3089.1 ± 402.7	3208.9 ± 436.7	3273.5 ± 493.8	3331.1 ± 424.1	3192.3 ± 437.6		< 0.001
Length (cm)	49.21 ± 1.75	49.53 ± 1.91	49.46 ± 3.40	49.93 ± 1.87	49.47 ± 2.02		< 0.001
Head (cm)	33.24 ± 1.21	33.55 ± 1.42	33.78 ± 2.35	33.90 ± 1.32	33.51 ± 1.47		< 0.001
MTHFR A1298C							0.524
AA	2376 (71.5%)	214 (6.44%)	70 (2.11%)	663 (19.95%)	3323 (64.35%)		
AC	1417 (71.06%)	153 (7.67%)	40 (2.01%)	384 (19.26%)	1994 (38.61%)		
CC	219 (73.74%)	19 (6.4%)	3 (1.01%)	56 (18.86%)	297 (5.75%)		
MTHFR C677T						0.324	
CC	2130 (71.4%)	199 (6.67%)	52 (1.74%)	602 (20.18%)	2983 (57.77%)		
CT	1558 (71.57%)	159 (7.3%)	46 (2.11%)	414 (19.02%)	2177 (42.16%)		
TT	324 (71.37%)	28 (6.17%)	15 (3.3%)	87 (19.16%)	454 (8.79%)		
Hcy	7.17 ± 1.54	7.11 ± 1.54	7.28 ± 1.75	7.08 ± 1.52	7.13 ± 1.56	0.295	

Data are shown as mean ± SD or n (%); BMI, body mass index; Hcy, homocysteine; MTHFR, methylenetetrahydrofolate reductase; *P*-values for differences among pre-pregnancy BMI categories were estimated using parametric or non-parametric methods, respectively, for continuous or categorical variables

### Association between maternal pre-BMI status and adverse pregnancy outcomes

The associations between the different pre-BMI groups and adverse pregnancy outcomes are presented in Table 2. The prevalence rates of GDM, GHT, CS, and PROM were 18.8%, 6.6%, 36.5%, and 24.1%, respectively. Based on the Chinese BMI classification, 3.2% of GDM cases (n = 34), 5.1% of GHT cases (n = 19), 3.0% of CS cases (n = 62), and 1.2% of PROM cases (n = 16) were included in the obese group, whereas the overweight group consisted of 11.6% of GDM cases (n = 122), 14.5% of GHT cases (n = 54), 9.4% of CS cases (n = 192), and 6.6% of PROM cases (n = 89). In the model adjusted for confounders, GDM, GHT, and CS were all positively associated with higher pre-BMI, whereas no significant association with PROM was suggested compared to the normal weight group. The ORs for overweight pregnant women were 1.73, 2.54, and 1.47 for GDM, GHT, and CS, respectively. Similarly, the risks in obese pregnant women were 2.13 times higher for GDM (*P* = 0.005), 3.53 times higher for GHT (*P* < 0.001), and 2.64 times higher for CS (*P* < 0.001) than for normal-weight pregnant women. Women who were underweight before pregnancy were less likely to experience GDM (OR = 0.72, 95% CI = 0.56–0.90), GHT (OR = 0.68, 95% CI = 0.46–0.99), and CS (OR = 0.66, 95% CI = 0.55–0.79).

**Table 2:** Association between maternal pre-BMI categories and adverse pregnancy outcomes

Pregnancy outcomes	BMI status	Case/Control	OR (95% CI)	*P*
GDM	Normal weight	753/3259	1 (ref)	
	Underweight	145/958	0.72 (0.57–0.90)	< 0.001
	Overweight	122/264	1.73 (1.31–2.29)	0.002
	Obesity	34/79	2.13 (1.33–3.41)	0.005
GHT	Normal weight	254/3758	1 (ref)	
	Underweight	45/1058	0.68 (0.46–0.99)	0.047
	Overweight	54/332	2.54 (1.72–3.75)	0.000
	Obesity	19/94	3.53 (1.96–6.36)	0.000
CS	Normal weight	1505/2507	1 (ref)	
	Underweight	290/813	0.66 (0.55–0.79)	< 0.001
	Overweight	192/194	1.47 (1.14–1.89)	0.003
	Obesity	62/51	2.64 (1.67–4.17)	< 0.001
PROM	Normal weight	989/3023	1 (ref)	
	Underweight	260/843	0.92 (0.76–1.11)	0.36
	Overweight	89/297	0.99 (0.73–1.34)	0.99
	Obesity	16/97	0.65 (0.76–1.16)	0.92

GHT were adjusted for maternal age, education level, parity, gestational age at delivery, infant sex, and homocysteine

GDM, PROM, and CS were adjusted for maternal age, education level, parity, and homocysteine

GHT, gestational hypertension; GDM, gestational diabetes mellitus; CS, cesarean section; PROM, premature rupture of membranes

### Association between maternal *MTHFR* gene polymorphism and adverse pregnancy outcomes

In this study population, the frequencies of the *MTHFR* C677T genotypes were 57.8% for CC, 42.2% for CT, and 8.8% for TT. Additionally, the A1298C AA, AC, and CC genotypes had frequencies of 64.4%, 38.6%, and 5.8%, respectively. The minor allele frequencies were 23.1% for *MTHFR* A1298C and 27.5% for *MTHFR* C677T in the control group, and all polymorphisms were consistent with HWE (*P* for HWE = 0.921 for A1298C and *P* for HWE = 0.259 for C677T). We then analyzed the associations between *MTHFR* polymorphisms in different models or genotypes and adverse pregnancy outcomes (Supplementary Tables S2 and S3). According to the results of the adjusted regression models, no significant association was observed between *MTHFR* gene polymorphisms and GDM, GHT, CS, and PROM (all *P* > 0.05).

### Association of pre-BMI and adverse pregnancy outcomes stratified by *MTHFR* A1298C or C677T dominant models

In the subgroup analysis, to explore the effects of genetic variations in *MTHFR* on the association between pre-BMI and pregnancy complications, we stratified pre-BMI into two subgroups according to the dominant model of *MTHFR* A1298C and C677T. (Tables 3 and 4). Although an interaction effect was suggested between pre-BMI and *MTHFR* A1298C AC + TT genotypes in PROM (*P* for interaction = 0.045), no statistical differences were observed between the genotypes.

**Table 3:** Association of pre-BMI and adverse pregnancy outcomes stratified by MTHFR A1298C dominant model

Pregnancy outcomes	BMI status	AA	AC + CC	*P* for interaction^#^
		OR2 (95% CI)	OR2 (95% CI)	
GDM	Normal weight	1 (ref)	1 (ref)	0.076
	Underweight	0.59 (0.44–0.81) *	0.95 (0.65–1.34)	
	Overweight	1.70 (1.19–2.45) *	1.83 (1.15–2.79) *	
	Obesity	2.11 (1.20–3.73) *	2.00 (0.87–4.60)	
GHT	Normal weight	1 (ref)	1 (ref)	0.201
	Underweight	0.64 (0.40–1.01)	0.64 (0.34–1.21)	
	Overweight	2.53 (1.57–4.09) *	2.28 (1.22–4.27) *	
	Obesity	2.20 (1.01–4.82) *	6.49 (2.67–15.79) *	
CS	Normal weight	1 (ref)	1 (ref)	0.073
	Underweight	0.76 (0.61–0.96) *	0.52 (0.39–0.69) *	
	Overweight	1.64 (1.17–2.30) *	1.26 (0.84–1.81)	
	Obesity	2.23 (1.29–3.86) *	4.18 (1.75–9.99) *	
PROM^#^	Normal weight	1 (ref)	1 (ref)	0.045*
	Underweight	0.93 (0.72–1.19)	0.87 (0.66–1.20)	
	Overweight	1.10 (0.75–1.61)	0.85 (0.51–1.39)	
	Obesity	0.67 (0.33–1.34)	0.60 (0.20–1.75)	

GHT were adjusted for maternal age, education level, parity, gestational age at delivery, infant sex, and homocysteine

GDM, PROM, and CS were adjusted for maternal age, education level, parity, and homocysteine

*P < 0.05;

^#^*P* for interaction was assessed by likelihood ratio test

**Table 4:** Association of pre-BMI and adverse pregnancy outcomes stratified by MTHFR C667T dominant model.

Pregnancy outcomes	BMI status		CC	CT + TT	*P* for interaction^#^
		OR2 (95% CI)		OR2 (95% CI)	
GDM	Normal weight	1 (ref)		1 (ref)	0.216
	Underweight	0.68 (0.49–0.93) *		0.76 (0.54–1.07)	
	Overweight	1.64 (1.10–2.42) *		1.84 (1.23–2.75) *	
	Obesity	1.90 (0.94–3.86)		2.40 (1.28–4.47) *	
GHT	Normal weight	1 (ref)		1 (ref)	0.474
	Underweight	0.77 (0.47–1.24)		0.51 (0.28–0.91)	
	Overweight	3.02 (1.82–5.01) *		1.82 (1.02–3.23) *	
	Obesity	4.72 (2.13–10.45) *		2.51 (1.07–5.84) *	
CS	Normal weight	1 (ref)		1 (ref)	0.582
	Underweight	0.59 (0.46–0.76) *		0.74 (0.58–0.96) *	
	Overweight	1.10 (0.76–1.55)		1.98 (1.38–2.85) *	
	Obesity	2.61 (1.32–5.17) *		2.70 (1.46–4.97) *	
PROM	Normal weight	1 (ref)		1 (ref)	0.184
	Underweight	0.85 (0.65–1.11)		0.97 (0.76–1.30)	
	Overweight	0.92 (0.59–1.42)		1.06 (0.69–1.61)	
	Obesity	0.50 (0.19–1.31)		0.77 (0.36–1.59)	

GHT were adjusted for maternal age, education level, parity, gestational age at delivery, infant sex, and homocysteine

GDM, PROM, and CS were adjusted for maternal age, education level, parity, and homocysteine

*P < 0.05;

^#^*P* for interaction was assessed by likelihood ratio test

For GDM, no significant association was observed in underweight pregnancy women in MTHFR A1298C dominant model, but a decreased risk of 0.59 (95% CI = 0.44–0.81, *P* = 0.016) was found in underweight pregnant women with the *MTHFR* C677T CC genotype. Pregnant women with a higher pre-BMI had a significantly higher risk of GDM, except for obese pregnant women with the A1298C AC + CC genotype and those with the *MTHFR* C677T CC genotype.

For GHT, we did not find a relationship between underweight and the two dominant models of *MTHFR*. Women who were overweight or obese were likely to experience GHT, and the highest odds ratio of GHT was observed in the obesity group for the A1298C AC + CC genotype (OR = 6.49, 95% CI = 2.67–15.79) and the C677T CC genotype (OR = 4.72, 95% CI = 2.13–10.45).

For CS, the effect direction and size were similar in the subgroup analysis, and the highest odds ratio for CS was observed in obese women with the A1298C AC + CC genotype (OR = 4.18, 95% CI = 1.75–9.99). Interestingly, an increased risk of 1.64 (95% CI = 1.17–2.30) for CS was observed in pregnant overweight women with A1298C AA genotype and 1.98 (95% CI = 1.38–2.85) in C677T CT + TT genotype.

### Association of *MTHFR* A1298C or C677T dominant models and adverse pregnancy outcomes stratified by pre-BMI classification

For A1298C polymorphism, we found that pregnant women with normal weight and A1298C AC + CC genotype had an increased risk of 1.18 (95% CI: 1.01–1.38) for CS and a decreased risk of 0.89 (95% CI: 0.51–1.55) for GDM, when the population was stratified by pre-BMI classification.

For C677T polymorphism, an increased risk of 1.74 (95% CI: 1.07–2.83) for CS was observed in pregnant women with overweight and CT + TT genotype, which was consistent with the subgroup analysis stratified by C677T dominant model (Supplementary Tables S4 and S5).

## Discussion

In this study, the prevalence of GDM, GHT, CS, and PROM in pregnant Chinese women was 18.8%, 6.6%, 36.5%, and 24.1%, respectively. According to the Chinese BMI classification [19], 19.7% of the participants were underweight, 6.9% were overweight, and 2.0% were obese. Compared to those in the normal weight group, pregnant women with a higher pre-BMI (≥ 24.0 kg/m^2^) were more likely to have GDM, GHT, and CS, while a low pre-BMI exerted protective effects on GDM, GHT, and CS. Moreover, no significant associations between *MTHFR* genotypes and maternal outcomes were observed. Among pregnant women in the obese group, an elevation in the risk of GHT (OR = 6.49) and CS (OR = 4.18) was observed among subjects with the A1298C AC + CC genotype compared to the AA genotype group. Our findings also suggested that overweight or obesity during pregnancy in women with the C667T AA genotype had a higher risk of GHT (OR = 4.72) compared to those with the CT + TT genotype.

Multiple risk factors can affect pregnancy health, including maternal nutritional status, lifestyle, age, environmental factors, placental changes, and epigenetic alterations [7]. Overweight and obesity, an increasing health problem, are generally considered important risk factors for adverse maternal outcomes, including GDM, preeclampsia, and CS [20–22]. A possible mechanism is that maternal overweight and obesity are related to the effects of oxidative stress, proinflammatory status, energy homeostasis, angiogenesis, and insulin insensitivity [23, 24]. In this analysis, 499 (8.89%) women were classified as overweight or obese before pregnancy based on the Chinese BMI standard, which is similar to previous studies in Lanzhou (10.75%) [25], Wuhan (6.63%) [26], and Taiwan (9.80%) [27]. In addition, our study suggested that higher pre-BMI in pregnant women was related to GDM, GHT, and CS, compared with those with normal weight, which are similar to those of previous studies on Western and Chinese populations. For instance, women who were obese before pregnancy were more likely to develop hypertensive disorders during pregnancy than those who had a normal pre-BMI (OR = 5.53, 95% CI = 4.28–7.13) [26]. A meta-analysis including 133 research reports was conducted to study the association between GDM and BMI, and it showed that the risk of developing GDM was 2.29-fold in overweight and 6.79-fold in obese pregnant women compared to normal-weight pregnant women [28]. Obese women in Ghanaian had a 2-fold increased risk for CS and a more than six-fold higher risk of pregnancy-induced hypertension [29]. A study conducted by Rudra et al. reported that being overweight before pregnancy is associated with the risk of preterm birth, and a weaker association was observed between spontaneous preterm birth and PROM [30], but we failed to find significant relationships between PROM and pre-BMI classification.

*MTHFR* C667T (rs1801133) and A1298C (rs1801131) are two of the most investigated SNPs. *MTHFR* C677T mutation causes the conversion of alanine to valine at position 677, leading to decreased MTHFR enzymatic activity [12]. Similarly, the A1298C polymorphism encodes glutamate to alanine substitution, leading to a decrease in enzyme activity, but to a lesser extent [31]. Emerging evidences suggested that MTHFR activity affected body fat storage, folate metabolism, and homocysteine methylation, which play important roles in human reproduction [32]. For example, a Chinese study showed that the *MTHFR* C677T TT genotype had a higher homocysteine level, and the recessive model of *MTHFR* C677T gene polymorphism was associated with preeclampsia renal function impairment [33]. The prevalence of CT and TT genotypes in patients with pregnancy-induced hypertension was higher than that in the control group, but no correlation was found with *MTHFR* A1298C mutations [34]. The maternal *MTHFR* A1298C CC genotype was associated with an increased risk of PE (OR = 1.80) compared to the AA genotype, while the paternal *MTHFR* C677T CT genotype was associated with an increased risk of GHT (OR = 1.60, 95% CI = 1.08–2.39) [35]. However, no associations were observed between MTHFR gene polymorphisms and adverse pregnancy outcomes in the present study. These inconsistent findings might be due to the small difference in homozygous mutations for *MTHFR* C677T and A1298C; 22 cases were A128C CC genotype and 43 cases were C677T TT genotype in overweight plus obesity group in our study. The small difference in homocysteine (Hcy) concentrations among the *MTHFR* genetic variations might be another reason. Only a slighter increasing of Hcy levels was found in C677T homozygous mutated subjects compared to CC and CT genetic subjects (TT: 7.07 ± 1.19 µmol/L vs. CT: 7.00 ± 1.23 µmol/L vs. CC:6.86 ± 1.27 µmol/L), and we failed to show significant promotions of Hcy concentrations among *MTHFR* A1298C genotypes.

In addition, *MTHFR* genetic variations have also exerted a regulatory effect on overweight or obesity. According to the results of a randomized controlled trial, intervention with folate consumption from natural foods for eight weeks had a beneficial effect on the reduction of inflammatory markers (TNF-α, IL-6, and IL1β) and Hcy in overweight and obese women with *MTHFR* C677T polymorphism TT genotype [36]. Renzo et al. conducted a dietary study in an Italian population and observed that participants with the C677T CT or TT genotype had a higher BMI, and the ratio of total body lean to total body fat was significantly lower in the mutated genotype group after dietary intervention [37]. However, the modification effects of MTHFR gene polymorphisms on the association between pre-BMI groups and adverse pregnancy outcomes. Our findings suggested that pregnant obese women with the A1298C AC + CC genotype were likely to experience GHT (OR = 6.49) and CS (OR = 4.26), whereas those with the C667T CC genotype had a higher risk of GHT (OR = 4.72). These associations presented in this study are difficult to interpret because there are few studies available regarding the interaction between *MTHFR* polymorphisms and being overweight or obese on the risk of GHT. Polymorphisms in *MTHFR* A1298C AC + CC genotype might decrease *MTHFR* enzyme activity, 5-methyl THF [38], and impaired production of Hcy (AA: 7.16 ± 1.53 µmol/L vs. AC + CC: 7.09 ± 1.59 µmol/L) in this study, which would explain the associations between the A1298C AC + CC genotype and GHT, but no association for maternal C677T CT + TT genotype and the risk of GHT. It might also due to small sample size of women with 54 cases of overweight and 19 cases of obesity for the subgroup analysis. On the other hand,similar ORs were observed for GDM and CS, which was consistent with the association between pre-BMI groups and the risk of GDM and CS mentioned above. Pregnant women had an increased risk of 2.11 (95% CI: 1.20–3.73) for GDM in obesity group and of 1.64 (95% CI: 1.17–2.30) for CS in the overweight group with *MTHFR* A1298C AA genotype and 2.40 (95% CI: 1.28–4.47) for GDM in the obesity group and 1.98 (95% CI:1.38–2.85) for CS in the overweight group with *MTHFR* CT + TT genotype. These findings are in parallel with those of studies on the interaction between being overweight/obese or *MTHFR* gene polymorphisms on GDM and CS risk. Maternal obesity is linked to an increased prevalence of CS and GDM [39]. A meta-analysis of the original epidemiological studies demonstrated that the susceptibility to GDM was higher in the C677T TT genotype, especially in Asians [40], which was further supported by a gene-nutrient interaction study based on a Chinese pregnancy cohort [41]. The difference between these two SNPs might be because A1298C non-mutated pregnant women with higher pre-BMI values are more likely to be homozygous or heterozygous mutants for the C677T polymorphism, which might be affected by the C677T mutation.

A comprehensive investigation including 5614 mother-infant pairs was performed to evaluate the association between pre-BMI, *MTHFR* polymorphisms, and adverse pregnancy outcomes. We also explored the influence of *MTHFR* polymorphisms on the association between maternal pre-BMI and risk of GDM, GHT, and CS. However, our study has several limitations. First, the data of pre-pregnancy BMI relies on self-reported weight and height, which may cause a possibility of confounding bias in data processing. Moreover, the sample size of pregnant women with a higher pre-BMI was relatively small. Second, several potential confounders, such as diet, physical activity, and other environmental exposures, were not controlled in our analysis model, which might have affected the reliability of our results. Third, only two common SNPs in *MTHFR* were included in our study, and future studies should focus on the modification effects of other variants of *MTHFR* gene polymorphisms.

## Conclusions

In summary, we conducted a retrospective study based on 5614 mother-infant pairs in Chinese population to explore the association of pre-BMI and *MTHFR* genotypes with the risk of adverse pregnancy outcomes, indicating that a higher pre-BMI (≥ 24.0 kg/m^2^) in pregnant women was positively related to the risk of GDM, GHT, and CS. We also found evidence that *MTHFR* polymorphisms could modify the association between maternal pre-BMI and risk of GDM, GHT, and CS. Pregnant women with obesity and *MTHFR* A1298C AC + CC or overweight with the C667T CC genotype had the highest risk for GHT. Our research could be used as a potential guideline for weight administration and gene detection before pregnancy to improve maternal outcomes in the Chinese population. Further investigations with larger sample sizes and prospective or multicenter designs are warranted to confirm our findings.

## Electronic supplementary material

Below is the link to the electronic supplementary material.

**Additional file 1:** Supplementary Figure and Tables.

## Data Availability

The datasets used and/or analysed during the current study are available from the corresponding author on reasonable request.

## List of abbreviations

BMI: Body mass index
CS: Cesarean section
CI: 95% confidence interval
GDM: Gestational diabetes mellitus
GHT: Gestational hypertension
Hcy: Homocysteine
HWE: Hardy-Weinberg expectation
MTHFR: Methylenetetrahydrofolate reductase
OR: Odds ratios
PROM: Premature rupture of membranes
