# Supplementary material for: Maternal pre-pregnancy BMI, MTHFR polymorphisms, and the risk of adverse pregnancy outcomes in pregnant women from South China: a retrospective cohort study
Source: BMC Pregnancy Childbirth. 2023 Apr 27;23:295. doi: 10.1186/s12884-023-05605-6 (PMC10134578; doi:10.1186/s12884-023-05605-6)
Supplement: Supplementary file 1 — Additional file 1: Supplementary Figure and Tables. [file 12884_2023_5605_MOESM1_ESM.docx]

**Supplementary Material**

**Maternal pre-pregnancy BMI, *MTHFR* polymorphisms, and the risk of adverse pregnancy outcomes in pregnant women from South China: a retrospective cohort study**

**Chunming Gu1†, Weixiang Wu1†, Kefeng Lai1, Huan Li1, Lihong Wu1, Weiming Lu1, Xiaolin Ruan1, Mingyong Luo1***

1Department of Clinical Laboratory, Guangdong Women and Children Hospital, Guangzhou, China

* Correspondence: Mingyong Luo; [luo-my@163.com](mailto:luo-my@163.com)

†These authors have contributed equally to this work.

Chunming Gu, [guchunming0119@163.com](mailto:guchunming0119@163.com); Department of Clinical Laboratory, Guangdong Women and Children Hospital, Guangzhou, China

Weixiang Wu, [samwoowu@163.com](mailto:samwoowu@163.com); Department of Clinical Laboratory, Guangdong Women and Children Hospital, Guangzhou, China

Kefeng Lai, [laikefeng9733@163.com](mailto:laikefeng9733@163.com); Department of Clinical Laboratory, Guangdong Women and Children Hospital, Guangzhou, China

Huan Li, [liwjy527@163.com](mailto:liwjy527@163.com); Department of Clinical Laboratory, Guangdong Women and Children Hospital, Guangzhou, China

Lihong Wu, [13929591071@163.com](mailto:13929591071@163.com); Department of Clinical Laboratory, Guangdong Women and Children Hospital, Guangzhou, China

Weiming Lu, [luweiming198809@163.com](mailto:luweiming198809@163.com); Department of Clinical Laboratory, Guangdong Women and Children Hospital, Guangzhou, China

Xiaolin Ruan, [yuetangxl@163.com](mailto:yuetangxl@163.com); Department of Clinical Laboratory, Guangdong Women and Children Hospital, Guangzhou, China

Mingyong Luo*, [luo-my@163.com](mailto:luo-my@163.com); Department of Clinical Laboratory, Guangdong Women and Children Hospital, Guangzhou, China

Number of pages: 6;

Number of figure: 1;

Number of tables: 5;


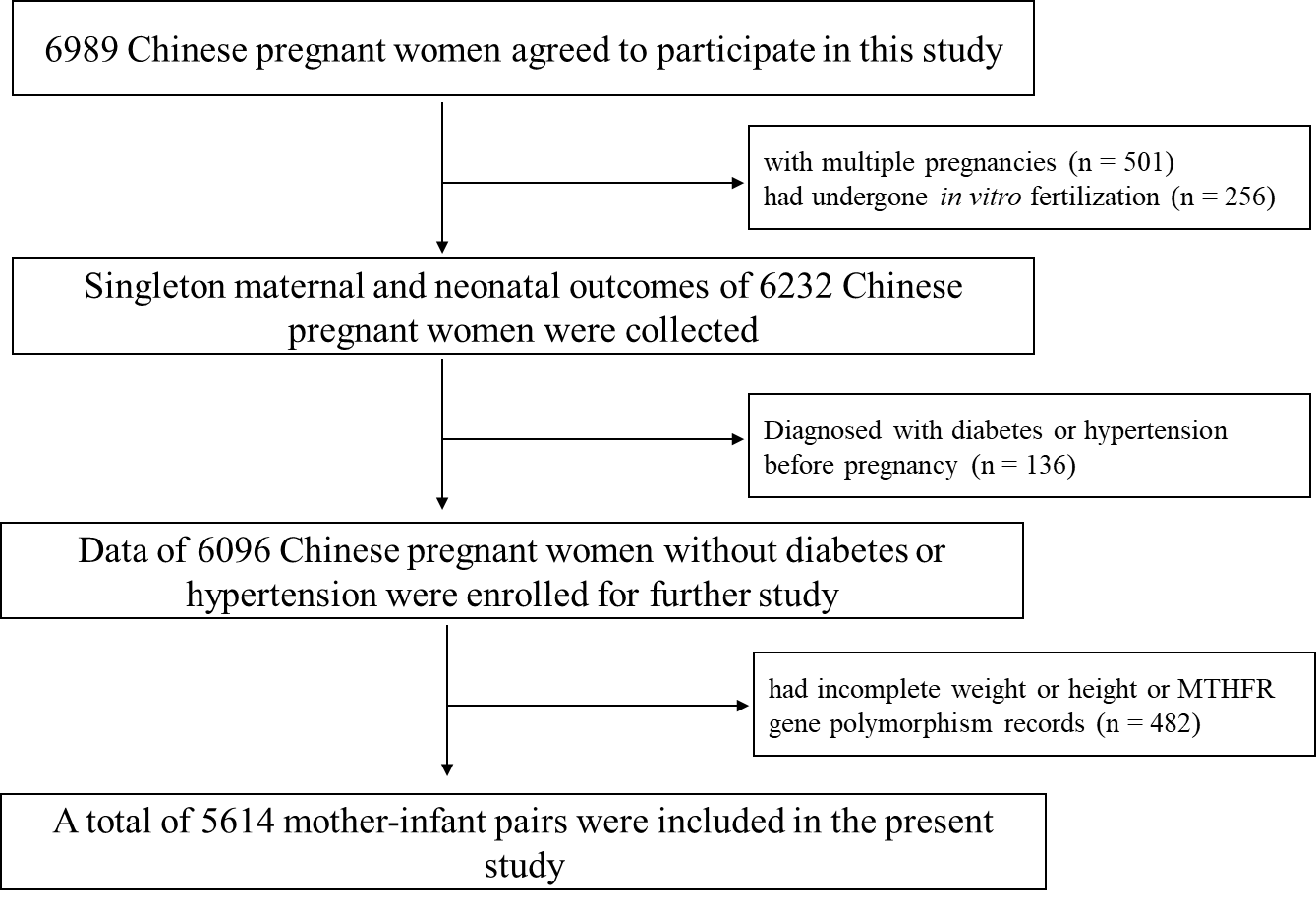


Fig. S1 Flow chart of this study

| TABLE S1. Primer sequences for MTHFR gene polymorphisms. | | |
| --- | --- | --- |
| Primer | forward sequence | reverse sequence |
| MTHFR C677T | 5’-CTCTTCTACCTGAAGAGCAAGTCC-3’ | 5’-CACTCCAGCATCACTCACTTTGT-3’ |
| MTHFR A1298C | 5’-CCGAAGCAGGGAGCTTTG-3’ | 5’-CGGTGCATGCCTTCACAA-3’ |

| TABLE S2. Association of MTHFR A1298C polymorphism and adverse pregnancy outcomes. | | | | |
| --- | --- | --- | --- | --- |
|  | MTHFR A1298C | case/control | OR2(95% CI) | *P* |
| GDM | AA | 654/2669 | 1 (ref) |  |
|  | AC | 352/642 | 0.87 (0.74-1.04) | 0.118 |
|  | CC | 48/249 | 0.79 (0.53-1.16) | 0.233 |
|  | Dominant Model |  | 0.86 (0.73-1.02) | 0.079 |
|  | Recessive model |  | 1.13 (0.95-1.33) | 0.168 |
|  | Additive Model |  | 0.83 (0.56-1.22) | 0.340 |
| GHT | AA | 238/3085 | 1 (ref) |  |
|  | AC | 119/1875 | 0.87 (0.74-1.04) | 0.118 |
|  | CC | 15/282 | 0.79 (0.53-1.16) | 0.233 |
|  | Dominant Model |  | 0.79 (0.61-1.02) | 0.068 |
|  | Recessive model |  | 1.22 (0.94-1.58) | 0.137 |
|  | Additive Model |  | 0.76 (0.41-1.42) | 0.392 |
| CS | AA | 1201/2122 | 1 (ref) |  |
|  | AC | 743/1251 | 1.13 (0.99-1.29) | 0.08 |
|  | CC | 105/192 | 0.92 (0.68-1.24) | 0.572 |
|  | Dominant Model |  | 1.10 (0.97-1.26) | 0.152 |
|  | Recessive model |  | 0.88 (0.77-1.01) | 0.061 |
|  | Additive Model |  | 0.88 (0.65-1.18) | 0.381 |
| PROM | AA | 823/250 | 1 (ref) |  |
|  | AC | 453/1541 | 0.87 (0.73-1.00) | 0.052 |
|  | CC | 78/219 | 0.98 (0.69-1.38) | 0.9 |
|  | Dominant Model |  | 0.87 (0.74-1.01) | 0.070 |
|  | Recessive model |  | 1.17 (1.00-1.36) | 0.051 |
|  | Additive Model |  | 1.04 (0.74-1.46) | 0.832 |

GHT were adjusted for maternal age, education level, parity, gestational age at delivery, infant sex, and homocysteine.

GDM, PROM, and CS were adjusted for maternal age, education level, parity, and homocysteine.

| TABLE S3. Association of MTHFR C677T polymorphism and adverse pregnancy outcomes. | | | | |
| --- | --- | --- | --- | --- |
|  | MTHFR C667T | Case/control | OR (95% CI) | *P* |
| GDM | CC | 568/2415 | 1 (ref) |  |
|  | CT | 401/1776 | 0.89 (0.75-1.05) | 0.174 |
|  | TT | 85/369 | 0.91 (0.68-1.22) | 0.536 |
|  | Dominant Model |  | 0.96 (0.84-1.10) | 0.586 |
|  | Recessive model |  | 1.04 (0.91-1.19) | 0.588 |
|  | Additive Model |  | 1.00 (0.78-1.27) | 0.976 |
| GHT | CC | 196/2787 | 1 (ref) |  |
|  | CT | 149/2028 | 0.87 (0.74-1.04) | 0.118 |
|  | TT | 27/427 | 0.79 (0.53-1.16) | 0.233 |
|  | Dominant Model |  | 1.02 (0.83-1.26) | 0.858 |
|  | Recessive model |  | 0.94 (0.76-1.17) | 0.601 |
|  | Additive Model |  | 0.88 (0.59-1.32) | 0.544 |
| CS | CC | 1071/1912 | 1 (ref) |  |
|  | CT | 802/1375 | 1.04 (0.91-1.19) | 0.57 |
|  | TT | 176/278 | 0.97 (0.76-1.22) | 0.775 |
|  | Dominant Model |  | 1.06 (0.95-1.18) | 0.324 |
|  | Recessive model |  | 0.98 (0.87-1.09) | 0.672 |
|  | Additive Model |  | 1.11 (0.91-1.35) | 0.295 |
| PROM | CC | 710/2273 | 1 (ref) |  |
|  | CT | 533/1644 | 1.13 (0.97-1.33) | 0.115 |
|  | TT | 111/343 | 1.12 (0.86-1.47) | 0.4 |
|  | Dominant Model |  | 1.04 (0.92-1.17) | 0.555 |
|  | Recessive model |  | 0.97 (0.85-1.10) | 0.611 |
|  | Additive Model |  | 1.02 (0.82-1.27) | 0.863 |

GHT were adjusted for maternal age, education level, parity, gestational age at delivery, infant sex, and homocysteine.

GDM, PROM, and CS were adjusted for maternal age, education level, parity, and homocysteine.

| TABLE S4. Associations of MTHFR A1298C polymorphisms and adverse pregnancy outcomes stratified by pre-BMI classification. | | | | | |
| --- | --- | --- | --- | --- | --- |
|  |  | AA | | AC+CC | |
|  | BMI status | case/control | OR (95% CI) | case/control | OR (95% CI) |
| GDM | Underweight | 86/577 | 1 (ref) | 59/381 | 1.21 (0.79-1.86) |
|  | Normal weight | 476/1900 | 1 (ref) | 277/1359 | 0.81 (0.67-0.98) |
|  | Overweight | 69/145 | 1 (ref) | 53/119 | 0.89 (0.51-1.55) * |
|  | Obesity | 23/47 | 1 (ref) | 11/32 | 0.75 (0.27-2.11) |
| GHT | Underweight | 29/634 | 1 (ref) | 16/424 | 0.71 (0.35-1.46) |
|  | Normal weight | 167/2209 | 1 (ref) | 87/1549 | 0.75 (0.56-1.03) |
|  | Overweight | 32/182 | 1 (ref) | 22/150 | 0.77 (0.37-1.60) |
|  | Obesity | 10/60 | 1 (ref) | 9/34 | 2.69 (0.77-9.39) |
| CS | Underweight | 183/480 | 1 (ref) | 107/333 | 0.86 (0.63-1.19) |
|  | Normal weight | 871/2289 | 1 (ref) | 634/1002 | 1.18 (1.01-1.38) * |
|  | Overweight | 110/104 | 1 (ref) | 82/90 | 0.84 (0.51-1.37) |
|  | Obesity | 37/33 | 1 (ref) | 25/18 | 2.23 (0.80-6.21) |
| PROM | Underweight | 32/631 | 1 (ref) | 20/420 | 0.89 (0.63-1.25) |
|  | Normal weight | 112/2264 | 1 (ref) | 83/1553 | 0.89 (0.74-1.05) |
|  | Overweight | 12/202 | 1 (ref) | 9/163 | 0.67 (0.36-1.22) |
|  | Obesity | 4/66 | 1 (ref) | 1/42 | 0.80 (0.20-3.11) |

GHT were adjusted for maternal age, education level, parity, gestational age at delivery, infant sex, and homocysteine.

GDM, PROM, and CS were adjusted for maternal age, education level, parity, and homocysteine.

**P* < 0.05;

| TABLE S5. Associations of MTHFR C677T polymorphisms and adverse pregnancy outcomes stratified by pre-BMI classification. | | | | | |
| --- | --- | --- | --- | --- | --- |
|  |  | CC | | CT+TT | |
|  | BMI status | case/control | OR (95% CI) | case/control | OR (95% CI) |
| GDM | Underweight | 77/525 | 1 (ref) | 68/433 | 0.95 (0.62-1.45) |
|  | Normal weight | 414/1716 | 1 (ref) | 339/1543 | 0.85 (0.70-1.03) |
|  | Overweight | 64/135 | 1 (ref) | 58/129 | 0.93 (0.54-1.59) |
|  | Obesity | 13/39 | 1 (ref) | 21/40 | 1.01 (0.39-2.62) |
| GHT | Underweight | 27/575 | 1 (ref) | 18/483 | 0.71 (0.35-1.41) |
|  | Normal weight | 128/2002 | 1 (ref) | 126/1756 | 1.01 (0.76-1.36) |
|  | Overweight | 31/68 | 1 (ref) | 23/164 | 0.65 (0.32-1.33) |
|  | Obesity | 10/42 | 1 (ref) | 9/52 | 0.47 (0.14-1.56) |
| CS | Underweight | 155/447 | 1 (ref) | 135/366 | 1.18 (0.86-1.61) |
|  | Normal weight | 797/1333 | 1 (ref) | 708/1174 | 0.92 (0.79-1.08) |
|  | Overweight | 91/108 | 1 (ref) | 101/86 | 1.74 (1.07-2.83) * |
|  | Obesity | 28/24 | 1 (ref) | 34/27 | 1.04 (0.41-2.59) |
| PROM | Underweight | 526/1604 | 1 (ref) | 463/1419 | 1.24 (0.89-1.72) |
|  | Normal weight | 41/158 | 1 (ref) | 48/139 | 1.07 (0.90-1.27) |
|  | Overweight | 7/45 | 1 (ref) | 9/52 | 1.24 (0.69-2.22) |
|  | Obesity | 136/466 | 1 (ref) | 124/377 | 2.28 (0.61-8.59) |

GHT were adjusted for maternal age, education level, parity, gestational age at delivery, infant sex, and homocysteine.

GDM, PROM, and CS were adjusted for maternal age, education level, parity, and homocysteine.

**P* < 0.05;
